# Age, sex—and what else? Rethinking priorities to close gaps in the HIV care cascade

**DOI:** 10.1002/jia2.70080

**Published:** 2026-02-09

**Authors:** Lise Jamieson, Sydney Rosen, Gesine Meyer‐Rath, Idah Mokhele, Nozipho Musakwa, Jeffrey W. Imai‐Eaton, Domonique M. Reed, Tsitsi Apollo, Dorlim Moiana Uetela, Daniel Shodell, Peter Ehrenkranz, Matthew P. Fox, the Closing The Gap Working Group

**Affiliations:** ^1^ Health Economics and Epidemiology Research Office, School of Clinical Medicine, Faculty of Health Sciences University of the Witwatersrand Johannesburg South Africa; ^2^ South African Centre for Epidemiological Modelling and Analysis (SACEMA) Stellenbosch University Stellenbosch South Africa; ^3^ Department of Global Health Boston University School of Public Health Boston Massachusetts USA; ^4^ Center for Communicable Disease Dynamics Department of Epidemiology Harvard T.H. Chan School of Public Health Boston Massachusetts USA; ^5^ MRC Centre for Global Infectious Disease Analysis School of Public Health, Imperial College London London UK; ^6^ HIV/AIDS and TB Department Ministry of Health and Child Care Harare Zimbabwe; ^7^ Instituto Nacional de Saúde Marracuene Mozambique; ^8^ Global Health, Gates Foundation Seattle Washington USA; ^9^ Department of Epidemiology Boston University School of Public Health Boston Massachusetts USA

**Keywords:** HIV treatment cascade, HIV testing, HIV treatment, viral suppression, UNAIDS targets, sub‐Saharan Africa

## Abstract

**Introduction:**

Many countries with high HIV burden have made substantial progress towards UNAIDS 95‐95‐95 targets and ending AIDS, but gaps in some sub‐populations hinder overall achievement, even as programmes face potentially diminished resources. While certain broad groups defined by age, sex or large geographic regions are commonly labelled as “high‐risk” for being out of care, most individuals within these groups are in care and virally suppressed. Characteristics beyond age and sex (e.g. behavioural, socio‐economic, smaller geographic areas) may differentiate those requiring targeted intervention strategies. Our *Closing The Gap* project aims to characterize unreached and disengaged sub‐populations for targeted HIV interventions across Mozambique, South Africa and Zimbabwe, countries selected for their varied target achievement and diverse populations. We discuss overarching themes from the first *Closing The Gap* workshop, convening government stakeholders, implementers, researchers and community representatives in February 2025.

**Discussion:**

Key themes emerged from the workshop: (1) the importance of considering absolute sub‐population size, alongside percentages, when assessing service gaps; (2) limitations of existing data and analytic paradigms beyond age‐and‐sex categories, highlighting the need for richer, contextual data linked to care cascade outcomes (e.g. clinical markers, mobility, socio‐economic circumstances) and analyses incorporating additional factors for identifying more granular sub‐populations; (3) need to identify individuals who do not require differentiated care to better prioritize resources to those not served by existing models; and (4) in the context of decreasing funding, the need to balance the cost and complexity of differentiated interventions with the feasibility and cost‐effectiveness of standardized approaches, including self‐selection strategies.

**Conclusions:**

It is critically important to generate more efficient strategies to close HIV care cascade gaps and sustain positive progress amidst potentially reduced future resources towards HIV. This may need a paradigm shift in service differentiation that specifically identifies sub‐populations most‐at‐risk of suboptimal outcomes, beyond age/sex categories, while efficiently balancing sub‐population size and proportionate risk. Data‐driven prioritization of cost‐effective interventions targeting the unreached and underserved is essential for sustaining progress in the evolving HIV response.

## 1 INTRODUCTION

In recent years, several African countries have reported achieving, or nearly achieving, UNAIDS 95‐95‐95 targets for HIV diagnosis, treatment and viral suppression [1], a critical step towards ending AIDS as a threat to public health. In most countries, however, certain sub‐populations still face gaps for each target, with shortfalls collectively impeding overall attainment. UNAIDS estimates that in 2024, of the 25.9 million people living with HIV (PLHIV) in sub‐Saharan Africa, 90% knew their status (22.3 million); 83% (19.9 million) were on antiretroviral therapy (ART), and 78% (18.6 million) were virally suppressed [1]. These estimates suggest that 10% of PLHIV do not know their status, 17% are not on ART and 22% are not virally suppressed. For specific countries, however, progress varies substantially in both directions. UNAIDS reports, for example, that an estimated 11% of PLHIV do not know their status in Mozambique [2], 33% of PLHIV are not on ART in South Africa [3] and only 9% of PLHIV in Zimbabwe are unsuppressed [4]. Many gaps are larger among specific sub‐populations, like young women aged 15–24 years living with HIV in South Africa, of whom about 44% are untreated [5], though these estimates rely on variable quality data, which may further deteriorate with recent funding cuts.

The persistence of service coverage gaps after more than 20 years of global and national investment [1]—and the recent international funding reductions that threaten the continuation of many existing programmes—suggests that, despite substantial progress in expanding coverage using existing interventions, new approaches will be needed to achieve overall targets. While many countries have streamlined services and reassessed priorities as a result of the funding cuts, limited opportunity remains for programmatic or data system investments, as available funding must now cover commodities previously supported by donors. Most countries have implemented focused HIV testing and treatment interventions for the roughly 6% of PLHIV who comprise key populations (men who have sex with men, female sex workers, transgender people, people who inject drugs and incarcerated people) [6, 7], though these bespoke services have been rapidly curtailed following shifts in US government funding priorities by the second Trump administration and non‐prioritization for domestic resources. Beyond this, most efforts at reducing care cascade gaps have focused on groups conceptualized solely by age and sex categories, and occasionally, by large geographic areas, including young women (15−24 years), men (15+ years), and children and adolescents [1]. These populations are all considered to need alternative, differentiated or enhanced service delivery models to ensure successful engagement and retention in care. Taking these groups together, few PLHIV remain who are regarded as “low risk,” resulting in little practical guidance for prioritizing interventions.

The problem with relying solely on age and sex to prioritize interventions for those outside of key populations, who comprise an estimated 94% of the total number of PLHIV in sub‐Saharan Africa, is that within any age‐and‐sex defined group, most PLHIV are in care and doing well with relatively standardized healthcare service delivery models. In Zambia, for example, young women (20−24 years) are considered one of the highest risk populations, yet two‐thirds (68%) are estimated to be on ART [8]. Similarly, addressing treatment gaps among men is a major HIV programme focus [9, 10], but across sub‐Saharan Africa, while treatment coverage among men lags that of women, the large majority (72%) of men are on ART [11]. Even adding the large geographic breakdowns sometimes used, such as province or district (e.g. “young women in province X”), does not alter this equation [12]. The challenge now is to identify which sub‐populations within these larger groups have distinct unmet needs, and to pair them with appropriate interventions.

Who might such sub‐populations be? Factors that could further distinguish sub‐populations with unmet care needs likely include behavioural, clinical, socio‐economic, household, community, facility and/or local or micro‐level geospatial characteristics. These characteristics are more nuanced than what is currently captured in large‐scale surveys or routine medical records. For example, in South Africa, a 2021 study found that among adolescent girls and young women with HIV, HIV status knowledge varied between those with (65%) and without (55%) a deceased parent [13]. A survey collected in 12 countries between 2015 and 2018 found differences in the association between food insecurity and ART use, with food insecurity associated with lower ART use in three countries (Ethiopia, Uganda and Namibia), higher ART use in one (Eswatini) and no association in the remaining eight. Additionally, characteristics can change over time, making their measurement and analysis more complex; our group have undertaken analyses to explore these shifts [14, 15, 16]. Behavioural, professional and identity characteristics have long been used to define HIV key populations, but they have not been applied beyond these groups. A recent review of strategies to improve HIV care cascade outcomes highlighted that studies have often failed to report on who benefited and why [17]. A consensus statement from the International Epidemiology Databases to Evaluate AIDS (IeDEA), a consortium of clinical cohort studies, proposed investment in community‐led surveillance, integration of routine data systems and real‐time digital tools [18] to enable more nuanced identification of sub‐populations.

There is a need to define and characterize sub‐populations more meaningfully than solely by age and sex. Such information could inform the design of context‐specific interventions that account for the complex and intersecting determinants of care engagement. Since collecting large quantities of new data to identify high‐risk sub‐populations may not be feasible in view of the recent funding reductions [19, 20], innovative analysis of existing datasets might provide at least some insights in response to new questions.

In this commentary, we discuss lessons learned in a workshop aimed at defining sub‐populations that may benefit from enhanced HIV care and treatment interventions in Mozambique, South Africa and Zimbabwe, countries selected for their variation in target achievement and income levels.

## 2 DISCUSSION

The *Closing the Gap in HIV Target Achievement* project aims to identify and characterize sub‐populations in sub‐Saharan Africa whose needs are not currently adequately met by HIV programmes, beyond the standard demographic characteristics of age, sex and large geographic area, and to recommend policies and programmes to address these gaps. In February 2025, the Closing the Gap workshop, in Johannesburg, South Africa, brought together teams from South Africa, Zimbabwe and Mozambique consisting of government ministry of health officials (South African National Department of Health, Mozambique's Ministry of Health and Zimbabwean Ministry of Health and Child Care), community representatives from civil society organizations (South African National AIDS Council, Treatment Action Campaign, I AM THAT MAN), research experts and programme implementers (Anova Health Institute, Human Sciences Research Council, Population Services International, Clinton Health Access Initiative, Ezintsha, Palindrome Data, Wits Health Consortium, University of Cape Town, Harvard T.H. Chan School of Public Health, Instituto Nacional de Saúde Mozambique, AIDS Healthcare Foundation Zimbabwe, Centre for Infectious Disease Research in Zambia, Partners in Hope Malawi, Malawi Liverpool Wellcome Programme, International AIDS Society, World Health Organization). Each team presented current evidence on gaps in the HIV care cascade, the availability and use of data and modelling approaches to identify gaps, and existing interventions aimed at addressing them. Following these presentations, teams worked to define and prioritize specific sub‐populations that might be considered for targeted intervention efforts. Themes were identified through an iterative process that integrated pre‐workshop review of country‐specific programmatic documents, agenda setting, note‐taking, and guided discussions during the workshop and post‐workshop transcription, verification and synthesis.

Over the course of the workshop, several common themes emerged that we discuss here. Workshop details, including presentations, further discussion of themes and the participant list, are available at https://www.heroza.org/publications/closing‐the‐gap‐in‐hiv‐target‐achievement‐workshop‐report.

**Theme 1: Strategies to close gaps must differentiate between percentages and numbers**.

When selecting targeted interventions, it is important to consider the absolute numbers of people affected, in addition to—or even instead of—the percentage of those engaged in the care cascade. Sub‐populations with high percentages not in care (i.e. not diagnosed, not on treatment or not suppressed) do not necessarily represent the largest absolute cascade gaps because some of these sub‐populations are relatively small. In contrast, some sub‐populations appear to be doing better in terms of percentages; however, they comprise a large number of individuals out of care.

**Figure 1:**
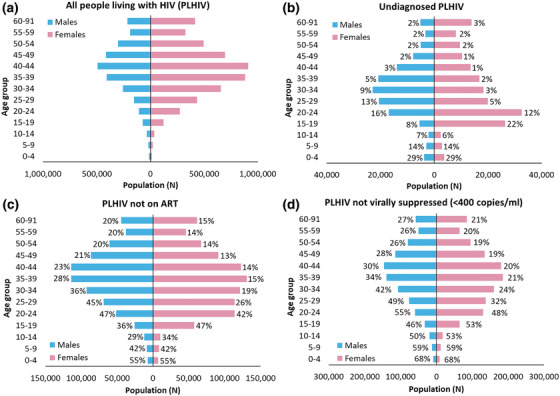
Number and percentage of people outside the care cascade for each 95 target by age and sex (percent out of each age/sex bracket), South Africa [5].

National estimates of care cascade gaps in South Africa by sex and age group exemplify this phenomenon (Figure 1) [5]. For example, the lower right pyramid, showing viral non‐suppression, indicates that in percentage terms, children (<15 years) have the highest probability of being unsuppressed (50−68%), but in absolute terms (X‐axis), they account for 78,400 individuals—3% of PLHIV not virally suppressed. In comparison, unsuppressed males and females aged 30–44 years account for 917,300 individuals—40% of the total unsuppressed population. Males in this age group, and females over the age of 30 years, are not commonly considered as “high risk” by policymakers [1, 21].

Age‐sex pyramids based on national estimates from the Thembisa model v4.8 depict gaps in the 95‐95‐95 targets, showing both the number (represented by the length of the bar) and the percentage not meeting the target (listed next to the bar) of each age and sex group. Panel A shows the number of people living with HIV, B shows the number (percent) not diagnosed among all PLHIV, C shows the number (percent) not on ART among all PLHIV and D shows the number (percent) not virally suppressed among all PLHIV. The length of each bar indicates the number of individuals by age and sex, with absolute numbers shown on the x‐axis. The percentage next to each bar indicates the percentage of all PLHIV in each age category.

Closing HIV care cascade gaps requires correlating heightened risk of suboptimal outcomes with absolute numbers—identifying groups large enough and sufficiently at risk to inform effective and cost‐effective targeting. To do so, research must go beyond focusing solely on basic demographic factors and consider multiple combined stratifications. Examining age, sex, large geographic area, and even just one additional factor—for example, marital or migrant status or profession—has the potential to improve targeting substantially. Optimizing intervention strategies requires correlating risk factors with the absolute number of individuals outside the care cascade, to identify where targeted efforts can yield the greatest benefit.

While large population groups might represent greater numbers of people outside the cascade in absolute terms, smaller sub‐populations may also warrant targeted interventions due to disproportionate transmission or mortality risk or equity considerations. For example, children living with HIV are more likely to be outside the care cascade than adults and were regarded by all country teams in our workshop as a high priority due to their high mortality risk. Yet, children comprise a much smaller percentage of the total population living with HIV than other groups. Improvements in the cascade for children will, therefore, not have as large an impact in closing the gap as would intervening for adult sub‐populations with higher numbers out of care. Workshop participants noted that countries may still choose to prioritize children for their elevated risk of mortality, even if the impact on overall country outcomes will be modest. This approach recognizes that HIV programming must balance epidemiological impact with other goals, particularly for vulnerable or marginalized populations.

**Theme 2: It is very hard to get beyond age and sex, in our data and in our thinking**.

Participants in the workshop were asked to use local data and modelling results to identify sub‐populations out of care in their countries beyond the large groups defined solely by age and sex. Meeting organizers worked hard to find information from anywhere in sub‐Saharan Africa that considered dimensions beyond age and sex to stratify and narrow groups out of care. To our disappointment, we found that such evidence barely exists in the published or grey literature. At the workshop, moreover, few participants were able to identify specific high‐risk sub‐populations within the larger age‐and‐sex groups, with the exception of key populations. Even when asked explicitly to speculate about which subgroups participants thought were most at risk, most reverted to populations defined by age and sex, with some exceptions. Patients with advanced HIV disease (AHD), reflecting delayed or disrupted care, were noted at the workshop to be at high risk of poor outcomes and may signal a critical point for identifying and targeting sub‐populations. Ideally, efforts should focus on reaching individuals at risk of AHD before this clinically significant stage is reached.

The CTG workshop thus underscored how accustomed those in our field are to thinking in terms of large age‐and‐sex groups, in addition to key population groups. We next asked each country team to start with the large age‐and‐sex groups they believed to be at highest risk of being out of care and then add “who also have Y risk factor” (e.g. “young women and girls who dropped out of school or have a poor socio‐economic background” was one sub‐population the Mozambique team suggested in response to this request), reflecting an intersectional lens.

A few examples in the literature demonstrate the potential of this more refined approach. In Zimbabwe, employment status and religious affiliation were associated with lack of diagnosis but not with engagement in ART or achievement of viral load suppression, suggesting that different factors may affect different stages of the care cascade [22]. Among young women in South Africa, socio‐economic status (SES) has been found to predict HIV care outcomes, with low SES predicting better cascade outcomes overall [13]. A simple factor, such as whether an appointment occurred before or after payday, predicted missed visit rates for working young men in South Africa [14].

These findings highlight the limitations of broad demographic targeting and the potential benefits of more nuanced approaches. This will require a change in how we think and analyse the care cascade, with stratification of populations by factors other than, or in addition to, age and sex. Estimating separate care cascades for employed versus unemployed persons, for example, which could potentially be created using existing survey and cohort study data, may help identify groups outside the care cascade that are both large enough to make a difference in cascade outcomes but more feasible for targeting interventions than simply “all working‐aged adults.” National household surveys, such as Population HIV Impact Assessment Surveys (PHIA), remain underutilized for defining alternative population segments, but stakeholders noted that they may offer opportunities to identify priority groups, and potentially, future surveys should prioritize collecting actionable risk data. Our project is undertaking a systematic review to identify characteristics associated with the cascade gaps, which may be used to inform subsequent data collection and targeting strategies [23]. Multilevel analysis of individual heterogeneity and discriminatory accuracy (MAIHDA), and other methodological approaches grounded in intersectionality, offer a way to identify high‐risk sub‐populations, supporting more nuanced and efficient HIV programme design [24].

**Theme 3: Focus on who is NOT disengaging from care, in addition to who is**.

Because available data may not indicate precisely who is disengaging from care, an alternative approach is to focus on identifying who is at low risk and/or already successfully engaged in care. By removing those individuals for whom current systems are working from the pool of people in need, programmes could allocate resources to those who most need support. This concept represents another important shift in thinking. For example, if even 40% of individuals in a priority sub‐population could be designated as securely engaged on ART, the resources available for the 60% with a greater risk of disengaging would be substantially greater, per individual. Differentiated HIV treatment delivery models that allow “established” ART clients to pick up medications in the community are an example of offering low‐resource‐intensity approaches, allowing healthcare workers to reallocate time to other needs [25, 26].

**Theme 4: Consider the trade‐offs between simple public health approaches and tailored care**

There may be instances in which the effort required to identify individuals with specific barriers to engaging in care will exceed its value in delivering improved population health outcomes and become burdensome for both clients and providers. For example, if substantial new data collection is required to categorize a male client as being in a sub‐population at high risk for disengaging, then the additional cost of offering proposed intervention(s) to all men, without differentiating individual risk, should be weighed against the cost of assessing risk. Texting visit reminders to all clients, for example, without differentiation by recipients’ pre‐assessed risk of missing an upcoming visit, may cost less than identifying and only messaging the high‐risk subsets. The latter is often done manually by health workers and can be time‐consuming. A programme‐level, potentially automated approach to stratification could reduce burden and improve efficiency. Some stakeholders proposed a “self‐needs assessment” strategy, in which clients choose which interventions they believe would assist them to achieve the next step in the care cascade. Offering choices brings its own complexities in practice [27], but it may unlock the potential for self‐selection to reveal individual health needs.

## 3 CONCLUSIONS

In an era of reduced donor funding for HIV care—and for collecting data about care and treatment—maintaining the progress that has been made, while continuing to close remaining gaps, will require a renewed focus on how to do more with fewer resources. This will require shifts in our thinking and a new look at existing datasets.

For both budgetary and technical reasons, expanding existing programmes and interventions may no longer be feasible. Even with effective differentiation, the global HIV response may be at a point of diminishing returns to investment—the point after which each additional percentage gain towards greater uptake and coverage will be more expensive and difficult to achieve. Better information about those not currently served, for whatever reason, is essential to determining which subgroups to prioritize for the biggest effect, where and how to reach them, and the most cost‐effective order in which to implement them.

## COMPETING INTERESTS

The authors have no conflicts of interest to report.

## AUTHORS’ CONTRIBUTIONS

Conceptualization of draft: SR, MPF and LJ.

Conceptualization of ideas, themes: SR, MPF, LJ, GM‐R, IM, NM, JWI‐E, DMR, TA, DMU, DS, PE and the Closing The Gap Working Group.

Evidence synthesis of existing modelling data: LJ and MPF.

Writing—original draft: SR, MPF and LJ.

Writing—review and editing: SR, MPF, LJ, GM‐R, IM, NM, JWI‐E, DMR, TA, DMU, PE and DS.

## Data Availability

The data that support the findings of this study are available through the Closing The Gap Workshop report, available at https://www.heroza.org/publications/closing‐the‐gap‐in‐hiv‐target‐achievement‐workshop‐report. Data presented on the size of the care cascade gaps were derived from the Thembisa model outputs available in the public domain at https://thembisa.org/downloads.
